# A segregation index combining phenotypic (clinical characteristics) and genotypic (gene expression) biomarkers from a urine sample to triage out patients presenting with hematuria who have a low probability of urothelial carcinoma

**DOI:** 10.1186/s12894-015-0018-5

**Published:** 2015-03-27

**Authors:** Laimonis Kavalieris, Paul J O’Sullivan, James M Suttie, Brent K Pownall, Peter J Gilling, Christophe Chemasle, David G Darling

**Affiliations:** Pacific Edge Ltd, Dunedin, New Zealand; Pacific Edge Diagnostics Ltd, Dunedin, New Zealand; Tauranga Urology Research, Tauranga, New Zealand; Department of Urology, Palmerston North Hospital, Palmerston North, New Zealand

**Keywords:** Macroscopic hematuria, Microscopic hematuria, Urine test, Urothelial carcinoma, Genotypic biomarkers, Gene expression, Phenotypic biomarkers, Triage, Clinical pathway, Urology

## Abstract

**Background:**

Hematuria can be symptomatic of urothelial carcinoma (UC) and ruling out patients with benign causes during primary evaluation is challenging. Patients with hematuria undergoing urological work-ups place significant clinical and financial burdens on healthcare systems. Current clinical evaluation involves processes that individually lack the sensitivity for accurate determination of UC. Algorithms and nomograms combining genotypic and phenotypic variables have largely focused on cancer detection and failed to improve performance. This study aimed to develop and validate a model incorporating both genotypic and phenotypic variables with high sensitivity and a high negative predictive value (NPV) combined to triage out patients with hematuria who have a low probability of having UC and may not require urological work-up.

**Methods:**

Expression of *IGFBP5*, *HOXA13*, *MDK*, *CDK1* and *CXCR2* genes in a voided urine sample (genotypic) and age, gender, frequency of macrohematuria and smoking history (phenotypic) data were collected from 587 patients with macrohematuria. Logistic regression was used to develop predictive models for UC. A combined genotypic-phenotypic model (G + P INDEX) was compared with genotypic (G INDEX) and phenotypic (P INDEX) models. Area under receiver operating characteristic curves (AUC) defined the performance of each INDEX: high sensitivity, NPV >0.97 and a high test-negative rate was considered optimal for triaging out patients. The robustness of the G + P INDEX was tested in 40 microhematuria patients without UC.

**Results:**

The G + P INDEX offered a bias-corrected AUC of 0.86 compared with 0.61 and 0.83, for the P and G INDEXs respectively. When the test-negative rate was 0.4, the G + P INDEX (sensitivity = 0.95; NPV = 0.98) offered improved performance compared with the G INDEX (sensitivity = 0.86; NPV = 0.96). 80% of patients with microhematuria who did not have UC were correctly triaged out using the G + P INDEX, therefore not requiring a full urological work-up.

**Conclusion:**

The adoption of G + P INDEX enables a significant change in clinical utility. G + P INDEX can be used to segregate hematuria patients with a low probability of UC with a high degree of confidence in the primary evaluation. Triaging out low-probability patients early significantly reduces the need for expensive and invasive work-ups, thereby lowering diagnosis-related adverse events and costs.

## Background

Hematuria, which is most often associated with causes such as benign prostatic enlargement, infection or urinary calculi, but is also symptomatic of urothelial carcinoma (UC), is estimated to occur in between 1 and 22% of patients in a general population [1,2]. Macroscopic (macro-) hematuria is characterized by a visible color change in the urine of patients, while microscopic (micro-) hematuria is defined more precisely as the presence of ≥3 red blood cells per high-powered field (RBCs/HPF) in three concurrently collected urine samples [2]. The overall prevalence of UC in patients with microhematuria has been reported to be approximately 4%, whereas several studies have consistently shown that the prevalence of UC is much higher in patients with macrohematuria, ranging from approximately 12–23% [2-6], yet up to four times as many patients with micro- versus macrohematuria present for urological evaluation [7]. Notably, given that recent changes to the American Urological Association (AUA) guidelines [2] have seen the threshold for asymptomatic microhematuria (AMH) lowered to ≥3 RBCs/HPF in a single sample, and even lower thresholds (≥1 RBC/HPF) have been proposed [8], a consequential increase in the number of patients with hematuria who will undergo a urological work-up to investigate potential UC and a corresponding increase in the overall clinical and financial burden of these patients on healthcare systems is expected.

Such hematuria-related referrals place a significant clinical burden on urologists, as all patients must undergo a full work-up to provide an often inconclusive diagnosis. Furthermore, the existing diagnostic tests – many of which are invasive or have high radiation loadings – can have a detrimental effect on patient quality of life (QoL), especially if the patient receives repeated cystoscopies as mandated in the current guidelines [2]. It has been reported that for cystoscopies performed without prophylactic antibiotics, 22% of patients had asymptomatic bacteriuria and 1.9% of patients developed a febrile urinary tract infection (UTI) within 30 days [9]. Other studies have also reported a high prevalence of macrohematuria, pain on voiding and transient erectile dysfunction in men following cystoscopy [10,11].

Healthcare systems also incur a significant financial burden as a result of patients with hematuria undergoing a full urological work up [12,13] and it has been concluded that urine cytology adds costs without offering any significant diagnostic benefit [14-16]. Consequently, integrating an accurate, non-invasive test into the primary clinical work-up of patients presenting with hematuria allows physicians to effectively triage patients with hematuria, thereby reducing the number of patients undergoing a full urological work-up and investigative cystoscopy for UC, and offers significant benefits to both patients and healthcare systems [15-19].

Several clinical prognostic characteristics, including age, gender, smoking history and degree of hematuria, are well-established as risk factors for UC in patients with hematuria [3,20-22]. Recently, several groups have attempted to develop models based on clinical prognostic characteristics to predict the risk of UC in patients with hematuria [20-22], but critically, these models offer limited accuracy and have largely focused on detecting patients with UC rather than ruling out patients who do not have disease. These detection-focused models have therefore been insufficient to reliably identify patients with disease during a primary evaluation, even if used in combination with urine cytology [20-22].

Despite the higher incidence of UC in patients presenting with macrohematuria, a number of studies show there is no significant difference in the distribution of UC by grade and stage in patients presenting with micro- compared with those presenting with macrohematuria [5,23-25]. Therefore, the AUA recommends that all patients with macrohematuria or AMH be referred to a urologist for a full urological work-up, as severity of hematuria is not sufficiently predictive for the presence of UC [2]. However, as patients with hematuria may only undergo limited urinalysis in a primary evaluation, consisting of cytology and in some cases imaging studies, such as ultrasound, a full urological work-up is often necessary to conclusively detect or rule out UC. While urine cytology is specified in current guidelines and routinely used in patients with suspected UC, cytology results are often inconclusive with atypical or suspicious findings and also suffer from a low diagnostic yield driven by a relatively high risk of false negative results for patients with UC-related hematuria [2,26,27]. Consequently, it can be difficult to rule out benign causes of hematuria, whether macrohematuria or AMH, during a primary evaluation, especially if UC-related hematuria is intermittent and appears to resolve following treatment for a benign cause [12].

A number of gene-based studies have set out to profile urinary biomarkers in patients with UC, and these biomarkers may be useful in their own right for detecting disease [28,29]. An opportunity also exists to triage out patients on the basis of their gene expression profile and clinical characteristics. Combining NMP22 enzyme-linked immunosorbent assay (ELISA) tests or a panel of gene markers with clinical characteristics has been shown to improve diagnostic accuracy compared with clinical characteristics alone, but these combined models have not yet delivered significant advances in overall diagnostic accuracy, especially when attempting to identify low-risk patients [30,31]. Nevertheless it is considered that incorporating clinical factors and specific gene expression into a combined algorithm is likely to provide the best guidance for diagnosing and managing patients with hematuria or UC [32].

Cxbladder Detect (Pacific Edge Ltd., Dunedin, New Zealand), a multigene test performed on unfractionated urine has previously been shown to be more sensitive than urine cytology and NMP22 for detecting UC in patients with macrohematuria [33] and more accurate than urine cytology, NMP22 and fluorescence *in situ* hybridization (FISH) in a comparative analysis (Breen, Kasabov, O’Sullivan, et al., unpublished observations). Cxbladder Detect uses quantitative reverse transcription polymerase chain reaction (RT-qPCR) technology to quantify five mRNA markers, four markers that are overexpressed in UC alongside a fifth marker that is elevated in non-malignant inflammatory conditions, and offers a high level of specificity and sensitivity when used to detect UC in patients presenting with hematuria [33]. It was hypothesized that an integrated model combining high-performance genetic biomarkers with phenotypic variables collected from the same patients will provide superior clinical resolution using high sensitivity (i.e. a low probability of a patient with UC receiving a false negative result), high negative predictive value (i.e. a high proportion of all negative results being true) and a high test-negative rate to enable the accurate triage of patients who have a low probability of UC. These genotypic and phenotypic variables when combined into a novel segregation model enable patients with hematuria who have a low probability of UC to be identified and triaged, as opposed to undergoing a full urological work-up.

## Methods

### Patient selection

A prospective sample of 695 patients has been analysed, where true clinical outcome was determined using a conventional clinical evaluation. The study sample consisting of an initial cohort of patients with hematuria was consented and sampled as previously described [33], where a consecutive series of 517 patients with a recent history of macrohematuria, aged ≥45 years and without a prior history of UC, were recruited prospectively from nine urology clinics in Australia and New Zealand. These patients were followed for three months for determination of UC status or alternative diagnosis, including upper urinary tract carcinoma [33] following multigene analysis of urine samples, with a positive UC diagnosis being based on cystoscopic appearance and histopathologic examination. The stage of disease was classified according to the TNM staging criteria determined by pathology and diagnostic imaging investigations and tumor grade was classified according to local pathology practice, using the 1998 World Health Organization (WHO)/International Society of Urological Pathology (ISUP) consensus classification [34].

Additional cohorts of 94 and 84 patients undergoing urological investigations following a macrohematuria event were subsequently recruited from two centers in New Zealand between March 2012 and April 2013 and included in the development of models. Centers were selected to participate on the basis of their previous experience participating in the initial study and their willingness to evaluate the Cxbladder Detect product within individual clinical settings.

An additional set of 45 patients presenting with microhematuria were consented and prospectively sampled prior to cystoscopic investigation for possible UC. Samples collected were used for further validation of the G + P INDEX, as set out below.

Eligibility criteria were similar to those of [33], except that patients aged ≥18 years and those who had previously undergone a cystoscopy to investigate UC that proved to be negative were eligible for enrolment. Furthermore, as in [33], patients exhibiting symptoms indicative of a UTI, or bladder or renal calculi, were excluded.

Ethical approvals were obtained from the New Zealand multiregion Health and Disability Ethics Committees, as required. All study participants provided informed consent prior to investigation.

### Urine sample collection and assessment

To provide gene expression data, a single mid-stream urine sample was collected from participants using the Urine Sampling System from Pacific Edge. Multigene analysis of samples from all studies was carried out in accordance with the standard operating procedure, as is used for the commercially available Cxbladder Detect multigenic test. All urine samples (4.5 mL) from the initial cohort were collected at a clinic prior to cystoscopy and transferred to a stabilization liquid via vacuum driven aspiration and sent to Pacific Edge within 48 hours. The samples were then stored at -80°C until required for batch analysis. Samples from the subsequent cohorts were collected in the same manner, but shipped to Pacific Edge at ambient temperature and processed within 7 days of sample collection in accordance with revised quality control (QC) limits and tolerance testing performed at the Pacific Edge diagnostic laboratory.

### Definitions

The term ‘phenotypic’ has been used to define clinical prognostic characteristics and to distinguish them from gene expression-based biomarkers that have been broadly defined as ‘genotypic’ variables.

### Statistical analysis

Univariate logistic regression was used to estimate the unadjusted (raw) log odds ratio (logOR) co-efficients for four binary phenotypic variables associated with UC: age, gender, smoking history and average daily frequency of hematuria events observed by the patient during the most recent hematuria episode (Hfreq; see Table 1). For microscopic hematuria, no events were observed, hence Hfreq = 0. Multivariate logistic regression on all four phenotypic variables was used to generate adjusted logOR co-efficients in the phenotypic model (P INDEX).

**Table 1:** **Definitions of binary phenotypic variables associated with UC and their corresponding scores**

**Phenotypic parameter**	**Score**	
	**0**	**1**
Gender	Female	Male
Age	<60 years	≥60 years
Smoking history	Never smoked	Current or past smoker
Hfreq	≤1 event/day	>1 event/day

G INDEX was developed using logistic regression to determine the association between UC and mRNA concentrations for the five Cxbladder Detect genes (*IGFBP5*, *HOXA13*, *MDK*, *CDK1* and *CXCR2*) in urine samples. A multivariate genotypic-phenotypic model (G + P INDEX) was generated using a combination of all nine variables from the G INDEX and P INDEX. These linear models determined the logOR from which the probability of a patient having UC can be derived.

The relative performance of each of model was illustrated in receiver operating characteristic (ROC) curves plotting the true positive rate versus the false positive rate when testing for UC, as determined by each model. Area under the curve (AUC) was used to summarize the performance of each model with an AUC approaching 1 deemed to be optimal.

To reduce potential bias when model estimation and prediction are performed on the same data set, a bias-corrected AUC was calculated for each of the three logistic regression models using bootstrap resampling [35]. The difference between the nominal AUC from the original sample and the average AUC from the bootstrap samples is an estimate of the sample bias and the nominal AUCs were adjusted accordingly. Bootstrap estimates of bias-corrected confidence intervals (CIs) were also obtained [36].

Furthermore, it was a design criteria for this clinical test that the performance characteristics of each model must exceed a threshold NPV of 0.97, with as high a sensitivity as possible with the further caveat of having a high test-negative rate. The test-negative rate is selected to provide a high clinical resolution when triaging out patients presenting with hematuria who have a low probability of having UC. Comparisons were made between the G INDEX, P INDEX and G + P INDEX and the performance of each model was determined in terms of sensitivity and NPV with a sufficiently high test-negative rate to provide an effective tool for triaging out patients with hematuria who have a low probability of UC.

## Results

### Sample demographics

**Figure 1:**
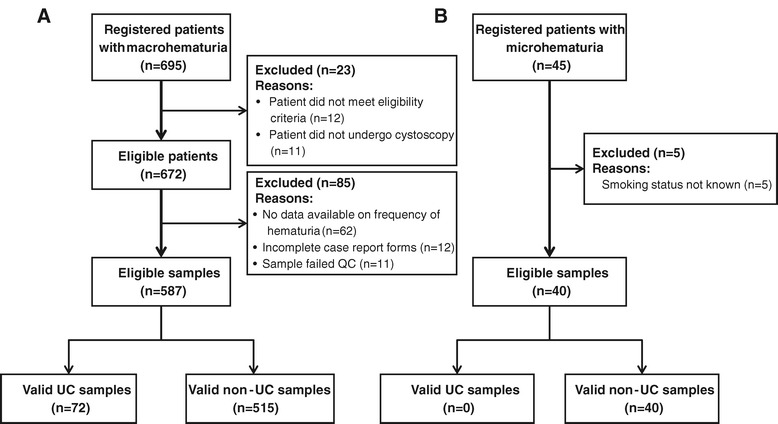
**Standards for Reporting of Diagnostic Accuracy (STARD) diagram for patient recruitment and enrolment.** Legend: **(A)** Patients with macrohematuria across all three cohorts in this study; **B)** patients with microhematuria included in this study.

Of the 695 patients with macrohematuria registered across the three cohorts, 23 were deemed to be ineligible and samples from a further 85 patients were excluded after enrolment due to the absence of sufficient data or samples failing to meet QC standards (see Figure 1A). In total, samples from 587 patients were available for modelling comprising 72 UC-positive and 515 UC-negative samples.

Of the 45 samples from patients with microhematuria provided, 40 were suitable for analysis with 5 patients deemed ineligible and excluded from the analysis (see Figure 1B). All 45 patients had received a full urological evaluation and clinical truth was confirmed as UC-negative. Full demographic data from both sample populations is presented in Table 2.

**Table 2:** **Sample population demographics for patients with macro- and microhematuria with complete data**

**Parameter**		**Patients with macrohematuria (N = 587), n (%)**	**Patients with microhematuria (N = 40), n (%)**
Age, years	<40	8 (1.4)	21 (52.5)
	40–49	47 (8.0)	
	50–59	107 (18.2)	
	60–69	143 (24.4)	19 (47.5)
	70–79	181 (30.8)	
	80–100	101 (17.2)	
Gender	Female	113 (19.3)	25 (62.5)
	Male	474 (80.7)	15 (37.5)
Smoking history	Never smoked	246 (41.9)	25 (62.5)
	Current or past smoker	341 (58.1)	15 (37.5)
Hfreq (events/day)	≤1	332 (56.6)	40 (100)
	>1	255 (43.4)	–
Tumor stage	Normal	515 (87.7)	40 (100)
	T1	16 (2.7)	–
	T2	11 (1.9)	–
	T3	2 (0.3)	–
	Ta	40 (6.8)	–
	Tis	3 (0.5)	–

### Relationship between phenotypic variables and risk of UC in patients with macrohematuria

**Figure 2:**
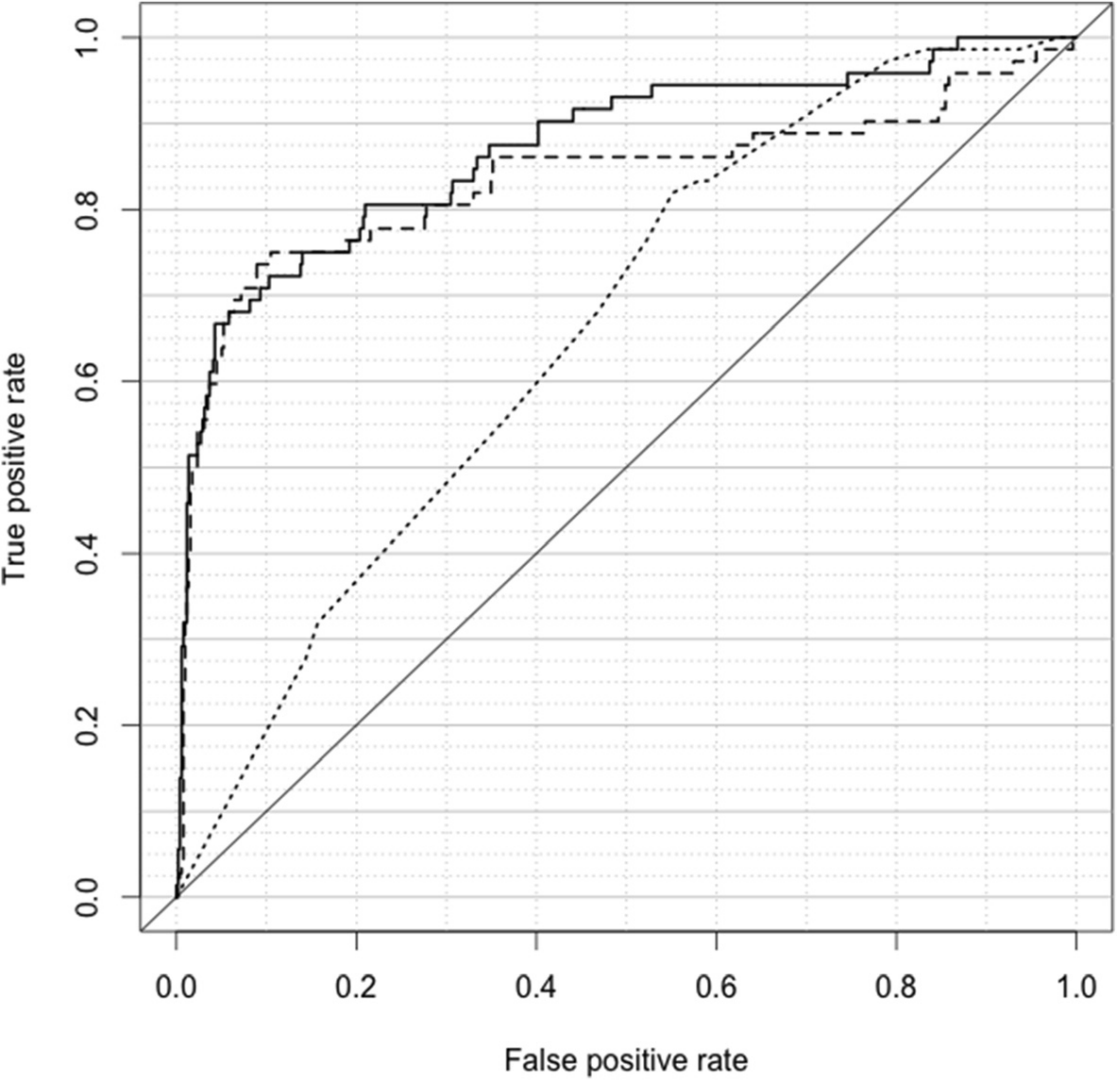
**ROC curves representing the three classification models.** Legend: P INDEX (dotted), G INDEX (dashed) and G + P INDEX (solid).

Univariate logistic regression analyses of each of the four binary phenotypic variables indicated that age ≥60 years, male gender, a history of smoking and a high frequency of macrohematuria were all associated with an increased risk of UC (Table 3). Adjusted logOR co-efficients were calculated in the multivariate logistic regression model.

$$ \mathrm{P}\ \mathrm{INDEX}=-3.78+\left(0.81\times \mathrm{Age}+0.46\times \mathrm{Gender} + 0.78\times \mathrm{Smoking}\ \mathrm{history}+0.59\times \mathrm{Hfreq}\right) $$

where each phenotypic variable is assigned a binary score of 0 or 1, as designated in Table 1, and the confidence intervals for the co-efficients are presented in Table 3. The bias-corrected estimate for AUC for the P INDEX is 0.66 (95% CI: 0.55–0.67; Figure 2).

**Table 3:** **Unadjusted and adjusted ORs for UC by phenotypic and genotypic factors for patients with hematuria**

				**Unadjusted OR (95% CI)**	**Adjusted P variable OR (95% CI)**	**Adjusted G + P variable OR (95% CI)**
**Phenotypic variables**		**Control (non-UC)**	**UC**			
Age, years	<60	151	11	2.30 (1.22–4.73)	2.24 (1.18–4.65)	1.89 (0.85–4.64)
	≥60	364	61			
Gender	Female	105	8	2.05 (1.01–4.75)	1.58 (0.76–3.72)	3.03 (1.12–9.36)
	Male	410	64			
Smoking history	Never smoked	227	19	2.20 (1.29–3.91)	2.19 (1.27–3.92)	2.67 (1.34–5.64)
	Current or past smoker	288	53			
Hfreq (average events/day)	≤1	300	32	1.74 (1.06–2.88)	1.80 (1.08–3.00)	1.76 (0.93–3.35)
	>1	215	40			
				**Unadjusted OR (95% CI)**	**Adjusted G variable OR (95% CI)**	**Adjusted G + P variable OR (95% CI)**
**Genotypic variables**						
*IGFBP5*				7.34 (4.59–12.33)	2.15 (1.03–4.58)	2.21 (1.03–4.83)
*HOXA13*				6.27 (3.92–10.34)	0.33 (0.13–0.83)	0.20 (0.07–0.56)
*MDK*				7.10 (4.73–11.10)	4.76 (1.74–13.62)	8.14 (2.64–26.60)
*CDK1*				7.80 (5.11–12.39)	3.47 (1.39–9.13)	2.59 (0.98–7.18)
*CXCR2*				1.69 (1.36–2.10)	0.65 (0.45–0.92)	0.69 (0.47–0.98)

Adjusted P INDEX, G INDEX and G + P INDEX variable ORs are the exponentiated co-efficients in the P INDEX, G INDEX and G + P INDEX, respectively.

### Relationship between genotypic variables and risk of UC in patients with macrohematuria

The G INDEX was estimated by logistic regression using the log mRNA concentrations of the five genes *IGFBP5*, *HOXA13*, *MDK*, *CDK1* and *CXCR2* in urine samples to predict UC occurrence.

$$ \mathrm{G}\ \mathrm{INDEX} = -6.22+\left(0.77\times IGFBP 5\;\hbox{--}\ 1.11\times HOXA 13+1.56\times MDK + 1.24\times CDK 1\;\hbox{--}\ 0.43\times CXCR 2\right) $$

The G INDEX gives a bias-corrected AUC of 0.83 (95% CI: 0.74–0.89; Figure 2).

### Relationship between genotypic and phenotypic variables and risk of UC in patients with macrohematuria

The five continuous genotypic variables were then combined with the four binary phenotypic variables to estimate the G + P INDEX using multivariate logistic regression.

$$ \begin{array}{l}\mathrm{G}+\mathrm{P}\ \mathrm{INDEX} = -8.46 + \Big(0.79\times IGFBP 5\hbox{--}\ 1.60\times HOXA 13+2.10\times MDK+0.95\times CDK 1\\ {}\hbox{--}\ 0.38\times CXCR 2\Big) + \left(0.64\times \mathrm{Age} + 1.11\times \mathrm{Gender} + 0.98\times \mathrm{Smoking}\ \mathrm{history}+0.56\times \mathrm{Hfreq}\right)\end{array} $$

The G + P INDEX gives a bias-corrected AUC of 0.86 (95% CI: 0.80–0.91).

### Comparison between G INDEX and G + P INDEX

There is overlap between the confidence intervals for the G INDEX and G + P INDEX, so a bootstrap version of a paired test was constructed by determining the difference in AUC for the G INDEX and G + P INDEX for each bootstrap sample. Ten thousand bootstrap samples with a sample size of n = 587 were generated by random sampling with replacement from the original 587 samples available for analysis. The resulting 95% CI for the difference between models was 0.01–0.08. Thus the probability that the true difference between the two AUCs is less than 0.01 is <0.025, indicating that there is a high likelihood of the AUC for the G + P INDEX being significantly greater than the AUC for the G INDEX.

### NPV and sensitivity of models

**Figure 3:**
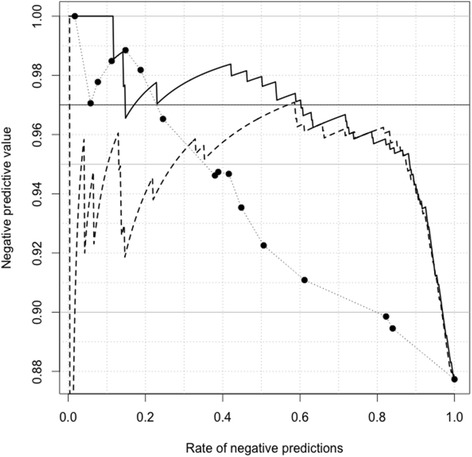
**NPV versus proportion of patients with hematuria testing negative according to the three classification models.** Legend: P INDEX (dotted), G INDEX (dashed) and G + P INDEX (solid) models. Note that the curve for the P INDEX is discrete as only 16 possible combinations of phenotypic variables are possible. The only possible values taken are indicated by the prominent points on this curve.

The G + P INDEX generated an NPV >0.97 over the range of test-negative rates from 0.2 to 0.7 and was almost always higher than the NPV for the G INDEX model (Figure 3). The G + P INDEX offered performance characteristics of sensitivity of 0.95 and NPV 0.98 when the test-negative rate was 0.4 (Table 4; Figure 3). In contrast, the G INDEX only achieved sensitivity of 0.86 and an NPV of 0.96 when the test-negative rate was 0.4 (Table 4).

**Table 4:** **Performance characteristics of each model when thresholds are set for varying test negative rates as determined on the macroscopic hematuria population**

**Threshold (logOR)**	**Test-negative rate (95% CI)**	**NPV (95% CI)**	**Sensitivity (95% CI)**	**Specificity (95% CI)**
**P INDEX**				
-2.54	0.25 (0.21–0.28)	0.97 (0.92–0.99)	0.93 (0.85–0.98)	0.27 (0.23–0.31)
-2.52	0.38 (0.34–0.42)	0.95 (0.91–0.97)	0.83 (0.74–0.91)	0.41 (0.37–0.45)
-2.39	0.42 (0.37–0.45)	0.95 (0.91–0.97)	0.82 (0.72–0.90)	0.45 (0.40–0.49)
-1.95	0.51 (0.47–0.54)	0.92 (0.89–0.95)	0.68 (0.56–0.78)	0.53 (0.49–0.57)
-1.93	0.51 (0.46–0.55)	0.92 (0.89–0.95)	0.68 (0.56–0.78)	0.53 (0.49–0.58)
-1.73	0.82 (0.79–0.85)	0.90 (0.87–0.92)	0.32 (0.22–0.43)	0.84 (0.81–0.87)
**G INDEX**				
-3.46	0.20 (0.17–0.23)	0.94 (0.88–0.97)	0.90 (0.80–0.95)	0.22 (0.18–0.25)
-3.23	0.30 (0.26–0.34)	0.95 (0.91–0.98)	0.89 (0.80–0.95)	0.33 (0.28–0.37)
-3.04	0.40 (0.36–0.44)	0.96 (0.92–0.98)	0.86 (0.77–0.93)	0.44 (0.40–0.48)
-2.86	0.50 (0.46–0.54)	0.97 (0.94–0.98)	0.86 (0.77–0.93)	0.55 (0.51–0.59)
-2.63	0.60 (0.56–0.63)	0.96 (0.94–0.98)	0.82 (0.71–0.90)	0.66 (0.62–0.69)
-2.41	0.70 (0.66–0.73)	0.96 (0.94–0.98)	0.78 (0.65–0.86)	0.77 (0.73–0.80)
**G + P INDEX**				
-4.02	0.20 (0.17–0.23)	0.97 (0.93–0.99)	0.96 (0.88–0.99)	0.22 (0.19–0.26)
-3.67	0.30 (0.26–0.33)	0.98 (0.94–0.99)	0.94 (0.87–0.99)	0.33 (0.29–0.37)
-3.33	0.40 (0.36–0.44)	0.98 (0.95–1.00)	0.95 (0.86–0.98)	0.45 (0.40–0.49)
-2.99	0.50 (0.46–0.54)	0.98 (0.96–0.99)	0.92 (0.83–0.97)	0.56 (0.52–0.60)
-2.71	0.60 (0.56–0.64)	0.97 (0.95–0.99)	0.86 (0.76–0.93)	0.67 (0.63–0.71)
-2.37	0.70 (0.66–0.73)	0.97 (0.94–0.98)	0.80 (0.70–0.88)	0.77 (0.73–0.80)

### Application of the G + P INDEX in patients with microhematuria

While the G + P INDEX was developed using data from patients with macrohematuria, its robustness was tested in a further 40 samples from patients with microhematuria (Hfreq = 0 for all microhematuria patients). A higher test-negative rate was expected in a microhematuria population as the incidence of UC is lower in this population, and using a test-negative rate of 0.4, 32 (80%) patients tested negative and would be correctly triaged out, therefore not requiring a full urological work-up for the determination of UC.

## Discussion

This study defines a clinical tool that offers clinicians and physicians the ability to effectively triage-out patients presenting with hematuria from the need to have a full urological work-up for the detection of UC. The study presents an internally validated genotypic-phenotypic model, G + P INDEX, with bootstrap-based CI estimates, that offers a combination of high sensitivity and high NPV (i.e. a low probability of an individual patient with UC providing a false-negative result and a high proportion of all negative results being true) that is not offered by models derived exclusively from genotypic or phenotypic data alone. This provides clinicians and physicians with a unique opportunity to triage out patients with both micro- and macrohematuria, in particular by identifying patients with a low risk of having UC who do not require a full urological work-up.

A high test-negative rate in the context of high sensitivity is an important consideration for an effective triage-out test that aims to direct patients with a low probability of UC away from a full clinical work-up [37]. Accordingly, at a test-negative rate of 0.4 the sensitivity of the G + P INDEX presented here maximizes both the sensitivity and NPV (0.95 and 0.98, respectively). This can be compared with the best fit selected from the genotypic model published in [33] (sensitivity = 0.82; NPV = 0.97) and is also comparable with the sensitivity and NPV of both cystoscopy (sensitivity = 0.89–0.98; NPV = 0.99) and virtual cystoscopy using computed tomography (CT) scans or magnetic resonance imaging (MRI) (sensitivity = 0.94 and 0.91, respectively) [38-40].

It is acknowledged that the population used to derive the G INDEX, P INDEX and G + P INDEX in this instance consisted of patients with macrohematuria. Therefore, the derived NPV values are only applicable to the macrohematuria population. The sensitivity of 0.95 (95% CI: 0.86–0.98) may, however, be applied across populations. Presuming that patients with and without UC are similarly distributed amongst the micro- and macrohematuria patient populations as depicted in [5,23-25], and that expected UC prevalence is 4% in the microhematuria population, a higher NPV and test-negative rate can be expected in the target microhematuria population.

By applying the G + P INDEX to the sample population of patients with microhematuria who do not have UC it was shown that 80% of the patients would have been triaged out on the basis of the result. Only 20% would be referred for a full urological work-up. This compares with conventional guidelines that would currently see all of the patients (100%) with microhematuria that cannot be attributed to a benign cause undergoing a full urological work-up, incurring significant unnecessary costs and negatively impacting patient QoL.

Severity of hematuria is correlated with the probability of a patient having UC, but not the stage or grade of any tumor, and an estimated 96% and 77–88% of patients with micro- and macrohematuria, respectively, referred to a urologist will not have UC [2-6]. Consequently, avoiding potentially unnecessary urological work-ups for patients with hematuria has several benefits. Cystoscopy may be associated with adverse effects, such as pain on voiding, bleeding, UTIs, male sexual dysfunction and the anxiety that accompanies an inconclusive or unconfirmed UC diagnosis [9-11]. Most notably, this novel approach has the potential to reduce the burden on resources and the financial cost associated with a full urological work-up on UC-negative patients. For example, in the UK, avoiding cystoscopy in patients with hematuria with an initial negative cytology and/or tumor biomarker test has been estimated to save approximately US$770 per patient (£483 per patient) evaluated [13]. The G + P INDEX described here provides an effective alternative to the use of urine cytology when used in a primary evaluation setting. This is particularly relevant in settings where primary evaluation is carried out by primary care physicians.

On this basis, if we assign an arbitrary ‘nominal cost’ of US$4,500 for each full urological work up, the total cost for working up 1,000 patients with microhematuria would approach US$4.5 million. In contrast, if 80% of patients with microhematuria are triaged out using the G + P INDEX at an arbitrary nominal cost of US$2,500, the total direct cost of testing and full urological work-ups for the remaining 20% of patients would total US$3.4 million. This provides a notional net saving in direct costs of approximately US$1.1 million per 1,000 patients with microhematuria.

The G + P INDEX and G INDEX, developed in this study, use the same genotypic biomarkers used in the genotypic model described in O’Sullivan et al. [33]. However, the G + P INDEX adds a further four phenotypic variables to enhance the ability to segregate patients who have a low probability of UC. The G + P INDEX uses a combinatorial method with a high sensitivity and a high NPV. By contrast, the genotypic model described in O’Sullivan et al. [33] optimizes the balance between sensitivity and specificity to calibrate the model calibrated for the optimal primary detection of UC in symptomatic patients (i.e. presenting with hematuria) who were undergoing a full urological work-up for suspected UC. The G + P INDEX has a significantly different clinical endpoint as no attempt is made to define or select patients with UC. Instead the aim is to confidently rule out those who do not have UC, and as such, all patients not segregated out would progress for a full urological work-up.

While several studies have previously sought to develop predictive models that consider phenotype when assessing the risk of UC in patients presenting with hematuria, the accuracy of phenotype-dependent models alone appears to be limited. For example, Loo et al. [21] prospectively investigated whether phenotypic parameters could be used to identify patients with microhematuria who may not have required a urological referral and full work-up and concluded that age, male gender and a recent diagnosis of macrohematuria were significant predictors of UC. A history of smoking and >25 RBCs/HPF in a recent urinalysis were not statistically significant predictors of UC, in isolation, but even when included in their ‘Hematuria Risk Index’ to improve predictive accuracy, this index resulted in an AUC of 0.809 [21]. Interestingly, the phenotypic ORs in this study and those identified by Loo et al. are comparable, with overlapping 95% CIs for smoking history and gender, and while age, gender and smoking history have similar weightings in each model, the influence of the genotypic component of the G + P INDEX presented here is likely to account for the higher AUC [21].

Likewise, Cha et al. [20] reported that age, smoking history and degree of hematuria, but not gender, were significantly correlated with the presence of UC in patients with asymptomatic hematuria and used a multivariate model to develop a nomogram comprised of phenotypic and urine cytology data for predicting UC. As with Loo et al. [21], the reported phenotypic ORs are comparable to those reported here, but even after incorporating urine cytology into the nomogram, the AUC of 0.831 reported in [20] was lower than that of the G + P INDEX.

In another study, Tan et al. [22] retrospectively stratified patients with hematuria who had been referred to a specialist urology clinic into high- and low-risk groups using a nomogram derived from patient age, gender, smoking history and the degree of hematuria. While comparisons with this study must be made with caution given the high proportion of patients who were excluded due to an absence of data (80 out of 405 patients), the AUC of 0.804, sensitivity of 0.900 and NPV of 0.953 were all lower than the G + P INDEX described here.

Several attempts have also been made to improve the accuracy of phenotypic models by supplementing them with the results of urinary biomarker tests. When the nuclear matrix protein NMP22 point of care proteomic assay is used in isolation to detect UC it has a sensitivity of 0.557 and NPV of 0.968 [17]. Lotan et al. [41] published a multivariable algorithm comprising phenotypic factors, NMP22 and urine cytology with an AUC for predicting UC of 0.826 that was then prospectively validated with an AUC of 0.802 [31]. However, it is important to note that this model attempted to discriminate between high-risk patients who did and did not have UC, as opposed to maximizing sensitivity and NPV to triage-out patients with a low probability of UC.

The improved accuracy obtained with algorithms comprising both genotypic and phenotypic data have previously been demonstrated in breast cancer, in particular [42-45]. Likewise, Mitra et al. [30] used a combination of molecular markers and smoking intensity to calculate a multivariate model that was superior to routine clinicopathological parameters in predicting survival in patients with UC. However, the present study is the first to demonstrate that phenotypic risk factors can be combined with genotypic data to increase the accuracy of a model for separating patients with hematuria into categories requiring differential levels of urological follow up and clinical care rather than survivorship prediction.

When phenotypic data are combined with genotypic data in a model, the resolution of data is likely to impact the accuracy of the model. For example, smoking is a well understood risk factor for UC and is included in most phenotypic models for detecting UC. In Cha et al. [20], Tan et al. [22], Lotan et al. [31,41] and the current study, the binary discriminants never smoked and current/ex-smoker were used, whereas Mitra et al. [30] calculated smoking intensity on the basis of years of smoking and number of cigarettes smoked each day and Loo et al. [21] categorized smokers into never smoked, passive smokers, smokers who had ceased and current smokers. While it is known that the risk of UC increases substantially with exposure to smoking [46], arbitrarily defining phenotypic variables may limit the overall accuracy and utility of phenotypic models. In contrast, an interaction between a patient’s genotypic and phenotypic variables would not be unexpected. However, combining the impact of phenotypic factors and genotypic variables in a single tool improved the accuracy of the model described in this study. A similar principle also applies to describing hematuria phenotype. Patients presenting with micro- or macrohematuria are essentially on a biological continuum and have different likelihoods of having UC [2-6,21]. Accordingly, despite all patients with microhematuria in this study having a Hfreq score of 0, the severity of their hematuria, in combination with other phenotypic factors, is likely to be indirectly accounted for in the genotypic component of the G + P INDEX.

## Conclusions

In conclusion, the G + P INDEX reported here shows a significant opportunity to change clinical utility. G + P INDEX is able to accurately triage out patients who present to their clinician or physician with hematuria, who have a low probability of UC with a high overall test-negative rate, high level of sensitivity and high NPV. This model could be suitable for use by physicians to triage out patients who do not require a full urological work-up, thereby reducing the number of patients with hematuria requiring a full urological evaluation for UC, helping to maintain patient QoL and helping to reduce diagnosis-related costs.

## Abbreviations

AMH: Asymptomatic microhematuria
AUA: American Urological Association
AUC: Area under the curve
CI: Confidence interval
CT: Computed tomography
ELISA: Enzyme-linked immunosorbent assay
FISH: Fluorescence *in situ* hybridization
HPF: High-powered field
logOR: log odds ratio
Hfreq: average daily frequency of hematuria events during the most recent hematuria episode
ISUP: International Society of Urological Pathology
MRI: Magnetic resonance imaging
NPV: Negative predictive value
OR: Odds ratio
QC: Quality control
QoL: Quality of life
RBC: Red blood cell
ROC: Receiver operating characteristic
RT-qPCR: Reverse transcription quantitative polymerase chain reaction
STARD: Standards for Reporting of Diagnostic Accuracy
UC: Urothelial carcinoma
WHO: World Health Organization
